# Enhancing accessibility through nurse-led clinics in primary care: An integrative review of models of care

**DOI:** 10.1016/j.ijnss.2025.10.006

**Published:** 2025-10-22

**Authors:** Yajai Sitthimongkol, Manassawee Srimoragot, Weha Kasemsuk, Saovaros Meekusol, Pokkrong Pongpattanapisit, Pennapa Saenkla, Suebsarn Ruksakulpiwat

**Affiliations:** Faculty of Nursing, Mahidol University, Bangkok, Thailand

**Keywords:** Community health, Integrative review, Models of care, Nurse-led clinics, Primary care

## Abstract

**Objectives:**

This integrative review aimed to examine and synthesize existing empirical evidence on nurse-led clinics (NLCs) in primary care settings, with a focus on models of care implemented globally.

**Methods:**

The review adhered to PRISMA guidelines, with rigorous inclusion and exclusion criteria applied. A systematic search was conducted across the Cochrane Library, EMBASE, Medline via EBSCO, PubMed, ScienceDirect, Scopus, and bibliographic databases for studies published between 2014 and 2024. Eligible studies included original, peer-reviewed research focused on nurse-led or nurse-managed clinics. A convergent integrated synthesis approach and thematic analysis were employed to identify key models of care.

**Results:**

The search yielded 1,651 records; 13 studies met the inclusion criteria. Data synthesis revealed six distinct models of care implemented in community-based nurse-led clinics: Innovative Cognitive Care, Integrated Multidisciplinary Care, Community-Driven Underserved Population Care, Reproductive and Women's Health Innovation, Palliative Care Model, and Behavioral Health Integration.

**Conclusions:**

Nurse-led models of care are crucial for strengthening primary healthcare, particularly in underserved settings. Further research and policy support are needed to expand nurses' roles, enhance their competencies, and promote interdisciplinary collaboration for the delivery of sustainable and equitable health services.

## What is known?

• Nurse-led clinics (NLCs) are an established model in primary care aimed at improving accessibility, efficiency, and patient-centered care delivery.
• Internationally, NLCs have demonstrated success in enhancing Continuity of care, reducing hospital readmissions, and improving patient satisfaction, particularly in chronic disease management.
• There is growing recognition of the potential of NLCs to address healthcare workforce shortages, especially in rural and underserved communities.

## What is new?

• The findings highlight innovative models such as Integrated Multidisciplinary Care, Community-Driven Underserved Population Care, and Behavioral Health Integration, which address diverse health needs and improve equity in healthcare access.
• Recommendations emphasize expanding nurse competencies, allowing basic medication provision in nurse-led settings, and strengthening the role of nurses as coordinators to optimize care delivery and support health policy development.

## 1 Introduction

The increasing demand for accessible, high-quality healthcare services has led to the development of diverse care delivery models worldwide. Among these, nurse-led clinics (NLCs) have emerged as a pivotal innovation in primary care, aiming to enhance service efficiency [1,2], improve patient outcomes [3], and address workforce shortages [2,4]. NLCs are defined as healthcare service units primarily managed and operated by nurses, often with advanced practice or specialist training, who take leading roles in assessment, diagnosis, treatment, follow-up care, health education, and chronic disease management [5,6]. Internationally, NLCs have been successfully implemented in various countries, including the United Kingdom [[7], [8], [9]], Canada [10,11], Australia [3,12,13], and the Netherlands [7,8]. These clinics are shown to be a transitional pathway from hospital discharge to the community, improving care continuity, reducing hospital admissions, depressive symptoms, and increasing patient satisfaction, while alleviating burdens on physician-led services. Empirical evidence suggests that NLCs contribute significantly to improving the quality of care and optimizing health system performance through a patient-centered, cost-effective, and community-responsive approach [3,12,14].

The development of NLCs has gained attention in recent years as a response to growing demands in primary healthcare. In Thailand, the National Health Security Office and Thailand Nursing and Midwifery Council have been supporting NLCs since 2021. The Thai healthcare system, driven by the Universal Health Coverage (UHC) policy, has increasingly emphasized the role of nurses in delivering essential services, particularly in rural and underserved areas [15,16]. Several NLCs have been initiated within hospitals, community health centers, and academic institutions, aiming to address the needs of patients with chronic diseases and to enhance health promotion and prevention. However, despite notable progress, several challenges remain, including limitations in regulatory frameworks, interprofessional collaboration, and professional capacity development [15,16].

Models of care within NLCs vary across settings, reflecting differences in national health systems, population needs, and professional scopes of practice. While multiple studies have explored specific nurse-led interventions, such as diabetes self-management programs in Thailand [17], heart failure care led by advanced practice nurses [18], and hypertension management in China [19], there remains a gap in synthesizing the overarching models of care and evaluating their structural and functional characteristics in primary care.

Given these gaps, this integrative review aimed to examine and synthesize existing empirical evidence on NLCs in primary care settings. Strengthening nurse competencies and institutional support structures will be essential for advancing NLCs as a sustainable solution for equitable and effective primary care.

## 2 Methods

### 2.1 Review design

This integrative review followed the Preferred Reporting Items for Systematic Reviews and Meta-Analyses (PRISMA) 2020 guidelines [21,22].

### 2.2 Problem identification

Three questions guided this review. 1) What models of NLCs have been implemented in primary care between 2014 and 2024? 2) What health outcomes have been reported in relation to NLCs in primary care? 3) What is the essential role of a nurse at a clinic?

### 2.3 Identify relevant studies

This review includes studies published between 2014 and 2024. The literature search was conducted across six databases: Cochrane Library, Embase, Medline via EBSCO, PubMed, Science Direct, and Scopus, using the following MeSH terms: (“nurse-led clinic∗” OR “nurse led clinic∗“ OR “nurse-managed clinic∗” OR “nurse practitioner clinic∗” OR “community nurse” OR “nurse practitioner”) AND (“primary health care” OR “primary care” OR “community health” OR “community-based care”) AND (“model of care” OR “care model∗” OR “service delivery” OR “health services” OR “healthcare service∗”) (Appendix A).

The inclusion criteria were: 1) peer-reviewed articles, 2) original research, including quantitative, qualitative, and mixed-method studies, 3) full-text articles published in English, and 4) studies focused on NLCs or clinics managed by nurses. The exclusion criteria were: 1) pilot studies, case studies, and quality improvement studies, 2) nursing home care, and 3) community care centers.

This review followed the PICO framework. The population (P) includes individuals receiving care services from nurse-led or nurse-managed clinics. The intervention (I) focused on nurse-led clinics in primary health care and community settings. The comparison (C), when applicable, was usual care. The outcomes (O) examined satisfaction and health outcomes. Four reviewers conducted the literature selection process to ensure methodological rigor and transparency. Y. Sitthimongkol and M. Srimoragot performed the initial database search and removed duplicates. Both independently screened titles and abstracts according to the inclusion and exclusion criteria. Y. Sitthimongkol, M. Srimoragot, P.Saenkla, and S. Ruksakulpiwat jointly conducted full-text screening and eligibility verification, resolving disagreements through discussion and consensus. Y. Sitthimongkol provided methodological oversight and validated the final list of included studies.

### 2.4 Quality appraisal

The methodological quality of the included studies was appraised using the Joanna Briggs Institute (JBI) Critical Appraisal Tools [23]. The JBI checklists were selected according to each study design, including tools for randomized controlled trials, quasi-experimental studies, qualitative studies, cross-sectional studies, and cohort studies. Each checklist consists of design-specific criteria used to evaluate methodological rigor, potential bias, and overall study quality. Two reviewers (Y. Sitthimongkol and S. Ruksakulpiwat) independently conducted the appraisal, and any discrepancies were resolved through discussion and consensus. The detailed results of the quality appraisal are presented in Appendix B.

### 2.5 Data analysis

A convergent integrated approach was employed to synthesize findings from the included studies, in accordance with the Joanna Briggs Institute (JBI) methodology for systematic reviews [20]. Thematic analysis was employed to systematically code, organize, and interpret the reported findings, enabling the identification of recurring patterns and categories across the studies.

## 3 Results

### 3.1 Search results

**Fig. 1:**
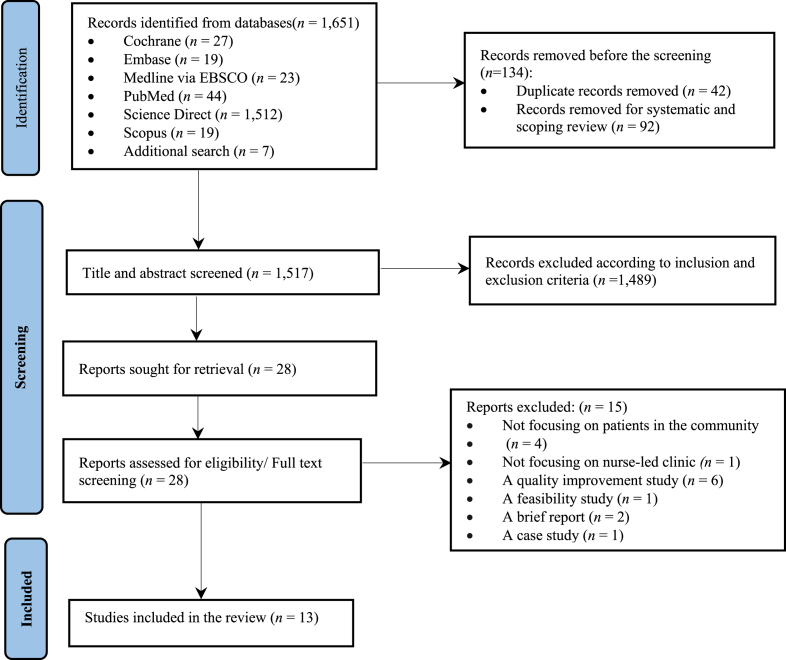
PRISMA flow diagram.

The search identified a total of 1,651 articles. Ultimately, 13 studies met the inclusion criteria and were incorporated into the final synthesis, as detailed in the PRISMA flow diagram [21] (Fig. 1).

### 3.2 Study characteristics

Data extracted from each eligible study included the following key variables: authors, year of publication, country, study setting, nomenclature, quality assessment, study design, sample size, clinic focus or disease area, study objective, main results, definitions of the community, nurse role, nurse competence, and suggestions for future research or policy implications (Appendix B).

The analysis of the 13 selected studies revealed several key trends regarding their characteristics. In terms of publication years, most studies were published in 2023 (30.77 %), followed by 2021 (23.08 %), while the least number of studies were from 2015 to 2020 (7.69 % each). Regarding geographic distribution, the majority of research was conducted across five countries: Australia, Canada, China, the United States, and the United Kingdom (23.08 % each). When categorized by continent, North America had the highest representation (46.15 %). Regarding research design, observational studies were the most common (46.15 %), followed by experimental studies, qualitative studies, and retrospective studies, each comprising 15.38 % of the total. The majority of studies used a sample size of 101–500 participants (53.85 %), while studies with 1–100 participants accounted for 15.38 %, and those with more than 500 participants made up 7.69 %. The most significant proportion of studies focused on mental health and neurological disorders (38.46 %), followed by research on chronic diseases and conditions, such as hepatitis C, diabetes, and urinary disorders (30.77 %). Studies related to sexual and reproductive health accounted for 15.38 %. The least studied populations were patients requiring palliative/end-of-life care and vulnerable groups, each representing 7.69 % of the studies.

The quality assessment indicated that, on average, 83 % of the methodological criteria were satisfied across all included studies. A summary of the quality appraisal results for each study is presented in Appendix B.

### 3.3 Definition of community

Included studies revealed that none of the research explicitly defined the term “community.” However, the studies referenced the concept of community in various aspects, particularly in terms of community engagement and healthcare service delivery models. Many studies focused on NLCs and programs designed to improve healthcare accessibility, particularly for underserved populations. The clinical environment varied depending on the context of each study. Many clinics operated in remote areas, where residents had limited access to healthcare services due to the long distance. Based on 13 studies, the classification of community settings for nurse-led clinics can be categorized as follows.

#### 3.3.1 Urban communities

These studies focused on clinics located in urban areas [[24], [25], [26], [27], [28], [29], [30], [31]]. Some of these clinics operated under nursing faculties within academic institutions [27,29,30].

#### 3.3.2 Remote areas

These studies examined clinics in remote locations [[32], [33], [34]]. Some research focused on proactive outreach services to improve healthcare access in these communities [26,35]. Additionally, telehealth was utilized to provide medical services remotely [32,36].

### 3.4 Roles of nurses

Nurses’ responsibilities include screening and assessing patients, recording clinical data, providing health education, promoting wellness, preventing diseases, administering vaccinations, ordering laboratory tests, managing patient care, and making referrals when necessary. Nurses also serve as coordinators between health professionals and community members, ensuring holistic and efficient healthcare delivery. Key responsibilities of nurses in NLCs include:

#### 3.4.1 Patient assessment and documentation

Nurses independently screen, assess, and document patient health conditions to ensure accurate clinical records and effective treatment planning.

#### 3.4.2 Health education

Nurses deliver direct education to patients on disease prevention, lifestyle modification, and self-care, and provide indirect care by equipping family members and caregivers with the knowledge needed to support the patient's ongoing health management.

#### 3.4.3 Health promotion

Nurses engage in community health initiatives, advocating for healthier lifestyles through awareness campaigns, workshops, and personalized consultations that address public health concerns.

#### 3.4.4 Prevention of diseases

Disease prevention efforts focus on communicable diseases such as HIV and sexually transmitted infections (STIs) by implementing screening, counseling, and risk-reduction strategies within the community.

#### 3.4.5 Non-pharmacological interventions

Nurses offer physical, mental, and social health interventions without relying on medication. These may include counseling, stress management techniques, rehabilitation services, and lifestyle coaching tailored to patient needs.

#### 3.4.6 Immunization services

Nurses are responsible for administering vaccines, ensuring proper immunization coverage for children, adults, and high-risk populations to prevent vaccine-preventable diseases.

#### 3.4.7 Care managers

Nurses coordinate and collaborate with physicians, psychiatrists, social workers, and other professionals to ensure integrated, patient-centered care. In this role, nurse coordinators manage care plans, facilitate referrals, and monitor progress to enhance treatment outcomes.

#### 3.4.8 Continuity of care and follow-up

Nurses monitor patient progress, laboratory results, treatment outcomes, and schedule follow-ups to track health improvements and address complications promptly. In NLCs, nurses collaborate closely with physicians to provide primary healthcare while also leading health education initiatives to improve self-care behaviors among community members. Additionally, nurses actively engage with community leaders, elders, and frontline health workers to prioritize and address public health challenges within diverse populations.

### 3.5 Qualifications of nurses

Most included studies did not specify the educational level of nurses working in nurse-led clinics. One study conducted in the United States indicated that the nurse practitioners in the clinic had obtained a Doctor of Nursing Practice (DNP) degree [36]. However, several studies emphasized the specialized training nurses must undergo before providing care in these clinics, ensuring competency in specific clinical areas [25,27,29,[32], [33], [34]].

### 3.6 Model of care in nurse-led clinics in the community

An analysis of 13 included studies revealed six distinct models of care implemented in NLCs within community settings (Appendix C). These models reflect the evolving roles of nurses and the diversity of healthcare services provided in response to the needs of various populations. The identified models—Innovative Cognitive Care, Integrated Multidisciplinary Care, Community-Driven Underserved Population Care, Reproductive and Women’s Health Innovation, Palliative Care Model, and Behavioral Health Integration—illustrate the capacity of NLCs to deliver adaptable, person-centered, and community-responsive care across a wide range of health priorities.

#### 3.6.1 Innovative cognitive care

The review identified implementing cognitive-focused therapeutic care models in NLCs as a practical approach to promoting cognitive health among older adults with cognitive impairments. A quasi-experimental study evaluated the outcomes of a nurse-led cognitive therapy program for older adults with Alzheimer’s disease. The results demonstrated a 30 % improvement in cognitive scores over a six-month period, indicating the effectiveness of a structured, nurse-delivered intervention in enhancing patients' quality of life [25].

Additionally, a study conducted in a nurse-managed cognitive assessment clinic affiliated with a university nursing faculty in Hong Kong found that approximately 44 % of patients did not adhere to the recommendations provided during the clinic visit. Non-adherence was particularly common among those with severe cognitive impairments and limited educational opportunities. Despite this, 96 % of patients expressed satisfaction with the support and guidance offered by the nurses [30].

#### 3.6.2 Integrated multidisciplinary care

The review revealed that integrated multidisciplinary care, particularly models emphasizing comprehensive, holistic care, was highly effective when implemented by nurses [24,26,28,31,35,36]. This model demonstrated significant improvements in both quality of life and overall health outcomes, especially among individuals with complex chronic conditions such as diabetes, heart failure, and hepatitis C. A study conducted in community clinics in Canada found that patients with hepatitis C who received holistic, team-based care had a treatment adherence rate of 74 % and a post-treatment virological cure rate of 96 % [24]. Another study found that integrated nurse-led multidisciplinary care also increased vaccination rates among homeless youth, access to HIV pre-exposure prophylaxis (PrEP), and expanded mental health services through telehealth [26].

In line with these findings, Fenton et al. [36] reported that nurse practitioner-led telehealth services in Maryland increased access to mental health care by 67 %. Moreover, telehealth services that involved nurse-led teams helped overcome transportation barriers and facilitated follow-up care, leading to a 35 % increase in appointment adherence among patients with depression.

#### 3.6.3 Community-driven underserved population care

The review identified several studies demonstrating the positive outcomes of NLCs that focus on underserved populations. For instance, a survey by Tominc et al. [26] conducted in Australia implemented the “Young People’s Health” initiative targeting over 400 homeless adolescents. The program included sexual health education, contraception counseling, and vaccinations administered by specialized nurses. Findings revealed that vaccination rates increased from 6.0 % to 38.8 %, while access to essential health information was improved. Moreover, a study conducted in Australia examined diabetes care in Indigenous communities and found a 30 % reduction in HbA1c levels, along with overall improvements in health outcomes. These outcomes were achieved through culturally sensitive screening and education efforts [33]. In Canada, another study emphasized the importance of community engagement in health service delivery. Nurses played a pivotal role in building trust between healthcare providers and patients, resulting in a 40 % increase in clinic utilization [35].

#### 3.6.4 Reproductive and women's health innovation

The review found that nurse-led reproductive and women’s health services in community settings play a critical role in significantly improving women’s access to healthcare. A study by Mazza et al. [32] evaluated the effectiveness of women's health clinics in rural and regional areas. In these clinics, specialized nurses provided services such as the administration of long-acting reversible contraception (LARC), telehealth consultations, medication-based abortion services, and screening for common conditions among women, including cervical cancer. The study reported that these NLCs increased access to women’s health services by up to 60 %, particularly among low-income populations.

#### 3.6.5 Palliative care model

The review found that community-based, nurse-led palliative care in resource-limited settings can significantly improve the quality of life for patients with advanced chronic illnesses. A qualitative study conducted in Liberia examined the experiences and perspectives of eight participants who received end-of-life care in a community clinic. The clinic’s care team consisted of eight palliative care-trained nurses and one physician assistant, delivering services to patients with terminal cancer. Participants expressed appreciation for the compassionate role of nurses, who not only provided clinical care but also coordinated effectively with local public health staff, ensuring smooth communication within the care team. This collaborative approach reduced wait times for health services compared to larger hospitals. Nurses also addressed patients’ multifaceted needs—physical, psychological, and economic—while being sensitive to cultural and religious beliefs [34].

#### 3.6.6 Behavioral health integration

The review revealed that NLCs integrating behavioral health into the management of chronic conditions, such as hypertension and depression, can significantly improve patients’ psychosocial self-management behaviors. For instance, a study by Holt et al. [29] demonstrated that mental health screening and care provided in community clinics led to a 25 % increase in treatment uptake among patients with coexisting hypertension and anxiety. Similarly, findings from Talley et al. [27] supported this model by showing that NLCs integrating behavioral health services reduced emergency department visits by 20 %.

## 4 Discussion

This study aimed to explore and evaluate innovative models of care implemented in NLCs, focusing on their impact on health outcomes, accessibility, and patient satisfaction. The review identified six models, each representing a unique approach to optimizing outcomes based on population needs. This discussion highlights the role of nurses, nurse-led services, and outcomes within models.

### 4.1 Innovative cognitive care

Cognitive care in NLCs emphasizes the pivotal role of nurses in supporting older adults with cognitive impairments through structured, patient-engaged programs [25,30]. In this model, nurses act as assessors, planners, and educators, delivering cognitive interventions and guiding both patients and caregivers to enhance quality of life. These findings suggest that nurse-led interventions can significantly improve cognitive functions and patient satisfaction [37,38]. The high satisfaction rates indicate that patients value the personalized care and expertise that nurses provide [37,38]. However, the noted challenges in adherence among patients with severe cognitive impairments call for the development of tailored strategies to enhance compliance. Future research should focus on identifying effective methods to support adherence in this population, potentially by exploring individualized approaches and integrating technology to facilitate the implementation of care plans. Additionally, expanding such nurse-led programs to diverse clinical settings could further validate their effectiveness and inform best practices for widespread adoption.

### 4.2 Integrated multidisciplinary care

The Integrated Multidisciplinary Care model, led by nurses in community clinics, improves outcomes for patients with chronic and complex conditions such as diabetes, hepatitis C, human immunodeficiency virus (HIV), lower urinary tract symptoms (LUTS), and heart failure [24,26,28,31,35,36]. Nurses play a central role in coordinating across disciplines, integrating the efforts of physicians, pharmacists, social workers, and allied health professionals to deliver comprehensive care. This approach enhances treatment adherence, accessibility, and chronic disease management, underscoring the importance of structured teamwork and shared decision-making in bridging care gaps [39].

These findings suggest that while nurse-led models effectively enhance coordination among healthcare professionals, sustained improvements require structural support, including policy recognition and resource allocation [40]. The interdisciplinary nature of this model ensures that patients receive holistic care tailored to their individual needs, yet challenges remain in optimizing workflows, clarifying role delineations, and promoting interprofessional education [41,42]. Future research should explore strategies to enhance interdisciplinary teamwork in NLCs, particularly in under-resourced areas where healthcare disparities persist. Additionally, longitudinal studies are necessary to assess the long-term sustainability and cost-effectiveness of these interventions across various healthcare systems. Investigating best practices for fostering collaborative care, particularly among high-risk populations with complex needs, will further refine the model’s effectiveness. Moreover, examining the role of digital health innovations, such as telemedicine and remote monitoring, in strengthening interdisciplinary collaboration could provide valuable insights for scaling up integrated nurse-led care models globally.

### 4.3 Community-driven underserved population care

Community-driven care models address underserved populations such as low-income groups, minorities, homeless individuals, and those in remote rural areas. Services are culturally tailored, recognizing that beliefs and community values strongly influence health behaviors and care decisions. Evidence shows that NLCs improve access and outcomes when grounded in community engagement. Programs addressing adolescent sexual and reproductive health [26,33,35,43] and culturally sensitive diabetes care for Indigenous populations [44] demonstrated improved participation, service utilization, and health outcomes. Nurses in this model act as frontline providers and community liaisons, fostering trust, delivering education, and collaborating with local leaders to adapt care to community needs [26,33,35]. Strengthening this model requires cultural education, active community participation, and adequate resources. Expanding nurse-led approaches through multisectoral collaboration could reduce disparities and promote sustainable healthcare delivery in underserved settings.

### 4.4 Reproductive and women’s health innovation

Nurse-led reproductive and women’s health models improve access to sexual and reproductive health, particularly in maternal health and family planning [32]. Integrating contraceptive counseling and medication-based abortion into community clinics further expands care for women in remote and underserved areas, reducing barriers and stigma [45]. Nurses in this model play a central role in counseling, providing preventive services, and delivering culturally appropriate care, thereby enabling them to utilize their professional expertise fully and support women’s health outcomes [32,45].

Furthermore, studies have reported that individuals seeking family planning counseling often face stigma and judgment in certain areas. Consequently, expanding outreach efforts and integrating family planning resources within primary healthcare clinics and community nursing clinics can significantly enhance access to reproductive health services [46]. The nurse-led care model is particularly well-suited for providing culturally sensitive care, especially for underserved women in various communities. By ensuring that services are accessible, non-judgmental, and aligned with the cultural beliefs and values of the populations they serve, community nursing clinics can contribute to improving reproductive health outcomes and overall well-being for women.

### 4.5 Palliative care model

Nurse-led palliative care clinics in resource-limited communities have been shown to significantly improve the quality of life for patients with terminal illnesses [34]. Nurses in this model act as coordinators, linking patients, families, and multidisciplinary providers to ensure continuity and individualized care [47]. Despite the global need, an estimated 40 million people require palliative care annually, with only 14 % receiving it at the end of life [48]—access remains limited, particularly in low-resource settings. Expanding nurse-led palliative care in primary care contexts could address this gap. To strengthen this model, nurses require adequate training and ongoing education to deliver culturally sensitive, high-quality care. Policy and system-level support are also crucial for sustaining and scaling nurse-led palliative care in underserved communities.

### 4.6 Behavioral health integration model

Integrating behavioral health into NLCs provides holistic care for patients with chronic conditions such as hypertension and depression. Evidence shows improvements in psychosocial outcomes, treatment engagement, and reductions in emergency visits and costly hospitalizations when behavioral health is embedded in nurse-led care [49]. Task-shifting approaches further demonstrate that general practice nurses, with proper training and supervision, can effectively deliver mental health services in the absence of specialists [50].

In this model, nurses play a central role in screening, counseling, and coordinating care, while also fostering treatment adherence and self-management. However, challenges remain, including workforce shortages and limited reimbursement for behavioral health services [49]. Strengthening this model requires investments in nurse training, supportive supervision, and policy changes to ensure adequate resources and funding. Expanding behavioral health integration within NLCs could advance equitable access to mental health care, particularly in underserved communities.

### 4.7 Clinical significance of the study findings

The clinical significance of this integrative review lies in its contribution to bridging research evidence with nursing practice in primary care. By synthesizing six distinct nurse-led models of care, the findings demonstrate that NLCs can play a transformative role in improving health outcomes, expanding accessibility, and addressing workforce shortages in both high-resource and resource-limited settings. These models demonstrate how nurses, when supported by appropriate training, policies, and institutional frameworks, can effectively address diverse health needs, ranging from cognitive care for older adults to reproductive health, palliative care, and the integration of behavioral health.

Digital health innovations further strengthen the reach of NLCs. Telehealth and remote monitoring improve Continuity of care in underserved areas. AI was not a focus of the included studies; however, its future integration into NLCs offers significant promise. AI could support early risk detection, individualized care planning, and workflow efficiency, enabling nurses to focus more on direct patient care.

Finally, the review demonstrates that policy support and institutional backing are indispensable for the sustainability and scalability of nurse-led models of care. Without regulatory recognition, adequate funding, and supportive infrastructure, the potential of these clinics to improve equity and efficiency in healthcare delivery cannot be fully realized.

## 5 Study limitation

This review has some limitations that need to be acknowledged. First, the inclusion of only English-language publications may potentially exclude relevant studies published in languages other than English. Second, the majority of the included studies were conducted in developed countries, which limits the generalizability of the results to low- and middle-income countries or settings with limited healthcare resources. Finally, most of the studies included in this review were observational in nature. While these studies provide valuable insights into current practices, they do not allow for firm conclusions about the effectiveness or causal impact of nurse-led care models.

## 6 Suggestion from the study

To further enhance accessibility, it is recommended that NLCs incorporate the provision of essential medications as part of their services. Enabling nurses, particularly nurse practitioners, to dispense and prescribe basic medications can reduce treatment delays, improve continuity of care, and address medication-related barriers, especially in underserved and remote areas. In the context of Thailand, policy support is needed to strengthen the role of nurse practitioners in providing basic medication as part of primary care services. This aligns with global practices and the direction of the Thai healthcare system under UHC, which emphasizes decentralization and increased access to frontline care. The findings underscore the importance of the nurse’s role as a care coordinator in NLCs. Nurses effectively bridge communication between patients and multidisciplinary teams, facilitate referrals, monitor treatment adherence, and ensure continuity of care. Strengthening this coordination role can significantly improve service integration, patient navigation, and health outcomes across community-based primary care settings.

## 7 Conclusion

Nurse-led models of care in primary care settings have been shown to improve healthcare accessibility and enhance patient health outcomes. These models enable nurses to assume expanded roles, significantly contributing to the delivery of effective and patient-centered care. To build a stronger evidence base, future research should investigate the effectiveness of nurse-led care across various primary care settings and among diverse populations. Such research is crucial for optimizing the utilization of nursing skills, informing healthcare policy, and promoting the broader adoption of nurse-led care, particularly in underserved or resource-limited areas where access to healthcare is limited.

## Data availability statement

The datasets generated during and/or analyzed during the current study are available from the corresponding author upon reasonable request.

## CRediT authorship contribution statement

**Yajai Sitthimongkol:** Conceptualization, Methodology, Validation, Formal analysis, Data curation, Writing – original draft, Writing – review & editing, Project administration, Supervision. **Manassawee Srimoragot:** Conceptualization, Methodology, Validation, Formal analysis, Data curation, Writing – original draft, Writing – review & editing. **Weha Kasemsuk:** Formal analysis, Validation, Investigation, Writing – review & editing. **Saovaros Meekusol:** Formal analysis, Validation, Investigation, Writing – review & editing. **Pokkrong Pongpattanapisit:** Formal analysis, Validation, Investigation, Writing – review & editing. **Pennapa Saenkla:** Formal analysis, Investigation, Data curation, Writing – original draft, Writing – review & editing. **Suebsarn Ruksakulpiwat:** Conceptualization, Methodology, Validation, Formal analysis, Investigation, Data curation, Writing – original draft, Writing – review & editing.

## Funding

This study was supported by the Health Systems Research Institute (HSRI), Thailand. The funding organization had no role in the study design, data collection, analysis, interpretation, or manuscript preparation.

## Declaration of competing interest

The authors declare there is no conflict of interest.
