# Supplementary material for: Enhancing accessibility through nurse-led clinics in primary care: An integrative review of models of care
Source: Int J Nurs Sci. 2025 Oct 22;12(6):593–600. doi: 10.1016/j.ijnss.2025.10.006 (PMC12684755; doi:10.1016/j.ijnss.2025.10.006)
Supplement: Multimedia component 1 [file mmc1.docx]

**专科护理门诊提升初级保健的可及性：整合性综述**

Yajai Sitthimongkol, Manassawee Srimoragot, Weha Kasemsuk, Saovaros Meekusol, Pokkrong Pongpattanapisit, Pennapa Saenkla, Suebsarn Ruksakulpiwat

【**摘要**】

**目的** 考察并整合初级保健环境中专科护理门诊（Nurse-led clinics, NLCs）的现有实证证据，重点关注全球范围内实施的护理模式。

**方法** 该综述遵循PRISMA指南，采用严格的纳入与排除标准。计算机检索the Cochrane Library、Embase、PubMed、ScienceDirect、Scopus等数据库，时间范围为2014至2024年间发表的文献。符合条件的研究需为聚焦护士主导或护士管理的诊所的原创同行评审文献。采用聚合综合分析法与主题分析法识别关键护理模式。

**结果** 共检索1,651篇文献，最终13篇文献符合纳入标准。数据整合揭示了社区专科护理门诊实施的6种护理模式：创新认知护理、整合多学科护理、社区驱动的弱势群体护理、生殖与女性健康创新、姑息护理及行为健康整合模式。这些模式展现了护士在提升医疗可及性、改善患者预后及响应社区特定健康需求方面多元且不断演进的角色。

**结论** 以护士为主导的护理模式对于加强基层医疗保健至关重要，尤其是在医疗资源匮乏的地区。还需要开展更多研究并提供政策支持，以扩大护士的职责范围、提升其专业能力，并促进跨学科合作，从而提供可持续且公平的医疗服务。

【**关键词**】 社区卫生；整合性综述；护理模式；专科护理门诊；初级保健

**通信作者：**Suebsarn Ruksakulpiwat，E-mail address: [suebsarn.ruk@mahidol.ac.th](mailto:suebsarn.ruk@mahidol.ac.th)
