# Supplementary material for: Enhancing accessibility through nurse-led clinics in primary care: An integrative review of models of care
Source: Int J Nurs Sci. 2025 Oct 22;12(6):593–600. doi: 10.1016/j.ijnss.2025.10.006 (PMC12684755; doi:10.1016/j.ijnss.2025.10.006)
Supplement: Multimedia component 2 [file mmc2.docx]

Appendix A. Search strategies

| Databases | Search strings |
| --- | --- |
| Cochrane Library | ("nurse-led clinic*" OR "nurse led clinic*" OR "nurse-managed clinic*" OR "nurse practitioner clinic*" OR "community nurse" OR "nurse practitioner") AND ("primary health care" OR "primary care" OR "community health" OR "community-based care") AND ("model of care" OR "care model*" OR "service delivery" OR "health services" OR "healthcare service*") |
| Embase | ('nurse led clinic'/exp OR 'nurse led clinic*':ti,ab OR 'nurse managed clinic*':ti,ab OR 'nurse practitioner clinic*':ti,ab OR 'community nurse':ti,ab OR 'nurse practitioner':ti,ab) AND ('primary health care'/exp OR 'primary care':ti,ab OR 'community health care':ti,ab) AND ('model of care':ti,ab OR 'care model*':ti,ab OR 'service delivery'/exp OR 'healthcare service*':ti,ab) |
| Medline EBSCO | ((MH "Nurse-Led Clinics" OR MH "Primary Health Care" OR MH "Community Health Nursing" OR TI ("nurse-led clinic*" OR "nurse managed clinic*" OR "nurse practitioner clinic*" OR "community nurse" OR "nurse practitioner")) AND (TI ("model of care" OR "care model*" OR "service delivery" OR "health service*" OR "healthcare service*"))) |
| PubMed | ("Nurse-Led Clinics"[MeSH] OR "nurse-led clinic*"[tiab] OR "nurse managed clinic*"[tiab] OR "nurse practitioner clinic*"[tiab] OR "community nurse"[tiab] OR "nurse practitioner"[MeSH Terms] OR "nurse practitioner"[tiab]) AND ("Primary Health Care"[MeSH Terms] OR "primary health care"[tiab] OR "primary care"[tiab] OR "community health"[tiab]) AND ("model of care"[tiab] OR "care model*"[tiab] OR "service delivery"[MeSH Terms] OR "healthcare service*"[tiab] OR "health service*"[tiab]) |
| Science Direct | ("nurse-led clinic" OR "nurse led clinic" OR "nurse-managed clinic" OR "nurse practitioner clinic" OR "community nurse" OR "nurse practitioner") AND ("primary health care" OR "primary care" OR "community-based care" OR "community health") AND ("model of care" OR "care model" OR "service delivery" OR "healthcare service" OR "health service") |
| Scopus | TITLE-ABS-KEY(("nurse-led clinic*" OR "nurse led clinic*" OR "nurse-managed clinic*" OR "nurse practitioner clinic*" OR "community nurse" OR "nurse practitioner") AND ("primary health care" OR "primary care" OR "community health" OR "community-based care") AND ("model of care" OR "care model*" OR "service delivery" OR "health service*" OR "healthcare service*")) |

Appendix B. Summary table of included studies

| - Authors, Year  - Study Setting /Country  - Nomenclature  - Quality assessment (%) | - Study design  - Sample size  - Clinic focus/area of disease | Study objective | Main results | Definitions of community | Nurse role | Nurse’s Competence | Suggestions for future research/policy implications |
| --- | --- | --- | --- | --- | --- | --- | --- |
| - Gagné et al., 2021 [24]  - Quebec, Canada  - Community-based NLC  - 54.5 % | - A clinical record-based prospective cohort study  - 171 patients infected with the hepatitis C virus  -Hepatitis C | 1. Examine the prevalence of patients initiating a hepatitis C treatment who: a) achieved sustained virologic response and  b) were adherent to their treatment; and  2. explore factors associated with adherence and sustained virologic response (achieved 12 weeks after the end of treatment) | 1. Report prevalence:  a) A total of 171 patients infected with the hepatitis C virus.  b) 126/171 (74 %) patients were adherent.  Among 156/171 (91%)  c) Patients with a hepatitis C virus RNA test post-treatment, 96% (*n* = 149) achieved sustained virologic response  2. Patients using illicit drugs were less likely to be adherent.  Patients who were less likely to achieve sustained virologic response were those who were non-adherent or who had a criminal record. | Urban areas | **Nurse role**  -deliver holistic patient-centered care and consolidate a network of outreach services for vulnerable people, connecting them with gastroenterologists and infectious disease specialists, as well as with other allied healthcare professionals (e.g., pharmacists, nutritionists, outreach workers). | Nurse practitioner: outreach, connect patients with specialists | -Research: A comparative impact of this nurse-led model of care for other vulnerable people on adherence, sustained virologic response, and healthcare costs is suggested.  -Policy: Nurse-led clinic should be considered to provide a safe and efficient coordination of interdisciplinary hepatitis C care. |
| - Yan et al., 2024 [25]  - China  - One memory clinic and four medical partnership communities  - 84.6 % | - Randomized wait-list controlled trial  - 144 participants  With Alzheimer’s Disease spectrum | To evaluate the effects of a nurse-led staged integral art-based cognitive intervention in older adults on  the Alzheimer’s disease spectrum. | 1. The intervention group showed greater improvement than the control group did in general cognitive functions  2. The intervention group showed significant improvement compared to the control group in language, memory, quality of life, sleep quality, and physical activity level at T1.  3. Statistically significant group differences  remained in sleep quality at the 6-month follow-up. | Provincial hospital clinics and medical partnerships in communities | **Nurse role**  -Nurse-led staged integral art-based cognitive intervention, a 16-week, 24-session (90 min per session). | -Trained professional nurses  - The nurses underwent 30 hr. of in-person training sessions with a qualified art therapist. During the intervention period, they received an additional 15 h of online training to  further enhance their knowledge and skills, address emerging issues, and promote adherence. | Research: Nurse-led staged integral art-based cognitive intervention program should be implemented in older adults with Alzheimer’s disease spectrum in other clinical settings to confirm the results. |
| - Tominc et al., 2023 [26]  - Melbourne, Australia  - NLC  - 75 % | A retrospective audit of electronic medical records.  - 400 participants aged 15 to  24 years, who had an encounter with the Young People’s Health Service (YPHS).  - YPHS is a co-located, NLC that aims to advance the health of young people experiencing homelessness.  The YPHS is  staffed by a nurse practitioner, advanced practice nurses, administrative staff, and rotating hospital medical officers. | 1) to identify the proportion of young people experiencing homelessness who are immunized against vaccine-preventable  Diseases.  2) to evaluate the impact of a nurse-led immunization program to improve vaccination coverage in this population. | 1. Integrating an  immunization nurse within a youth specialist homelessness service increased the percentage of young people who were up-to-date with routine vaccinations from 6.0 % (*n* = 24) to 38.8 % (*n* = 155).  2. The most common  The factor was disengagement from mainstream schooling (77 %, *n* = 307). Substance use was common, including intravenous (13 %, *n* = 52), and non-intravenous use (56 %, *n* = 223). | Young people experiencing Homelessness | **Nurse role**  The model reported is its **integration across multiple services** where the nurse can build strategic relationships, improve communication with multi-disciplinary professionals, and enhance health service experiences for the young people. | NP, Advanced Practice Nurses | Policy: NLC can improve vaccination coverage through co-located with homelessness providers. |
| - Talley et al., 2021 [27]  - Birmingham, Alabama, USA  - PATH and  Heart Failure Transitional Care Services for Adults clinics  -88.8 % | Academic-practice partnership: School of Nursing  and its partner, University of Alabama at Birmingham Hospital and Health System  520 patients qualified  250 patients are using the service  BH: alcohol intake, tobacco use, Depression, Anxiety, Mood Disorder | To describe an innovative model in which BH  Services are integrated into NLC for uninsured and underinsured patients. | Lower numbers of mental illnesses and  substance use disorders  Statistically significant  reduction in PHQ-9 or GAD-7 scores | The nurse-led PATH Clinic provides free care for uninsured patients with diabetes who are potential high utilizers of the University of Alabama at Birmingham Health System and are referred because of poorly controlled diabetes. | Nurse Coordinator | Psychiatric mental health nurse practitioner, psychiatrist, clinical social worker, and care coordinator, | Future initiatives should promote academic–practice partnerships to strengthen coordination between nursing schools and NLC, supporting integrated behavioral health services and workforce development. |
| - O’Byrne et al., 2021 [28]  - Canada  - Nurse-led PrEP- RN clinic  - 100 % | - A prospective cohort study  - 347  - An urban public health unit and community-based sexually transmitted infection (STI) clinic | To report the results of a nurse-led pre-exposure prophylaxis (PrEP) delivery service, specifically focusing on its effectiveness in increasing access to HIV prevention among high-risk individuals. | - Among 347 high-risk patients, 47 % accepted PrEP, with 69 % of eligible participants attending their intake visit and 66 % retained in care.  - Half of the participants continued PrEP, while the other half were lost to follow-up.  - The study confirms that nurse-led PrEP delivery is a viable and preferred strategy for accessing HIV prevention services. | An urban public health unit and community-based STI clinic that serves approximately 20,000 patient visits per year. | **A nurse-led task-shifting approach to HIV prevention through PrEP**  - **Nurse-Led Delivery**: Public health nurses are responsible for initiating and managing PrEP, shifting the task from physicians to nurses to increase access and efficiency.  - **Rapid Access and Community-Based Services**: The model emphasizes providing quick access to PrEP in a community setting, specifically within an STI clinic, to better serve high-risk populations.  - **Targeted Approach**: The care model focuses on individuals identified as high-risk based on clinical judgment and established criteria, ensuring that resources are directed to those most in need.  - **Accessibility**: By being located in a familiar and easily accessible area, the model aims to reduce barriers to care for individuals seeking HIV prevention. | - Pre-Exposure Prophylaxis–Registered Nurse  - N/A | Future research should assess the long-term effectiveness of nurse-led PrEP delivery and strategies to enhance access in underserved populations. Policymakers should support nurse-led interventions, ensure funding for PrEP services, and promote cross-training among healthcare providers. Community partnerships can also improve access for high-risk individuals. |
| - Holt et al., 2020 [29]  - USA  - Community Nursing Centers’ (CNCs)  - 75 % | Descriptive research  NA  BH Services | to share part of the 12-month formative program evaluation for this project that assessed the impact of the increased level of BH service integration on the types of psychosocial problems identified and treatments provided, and ratings of the primary care patients’ psychosocial health literacy, related self-management behaviors, and severity of psychosocial conditions. | The CNC Model and the AIMS Center Collaborative Care Model were shown to be the most utilized BH integration provider interventions. Omaha System outcome ratings showed statistically significant improvements  in psychosocial self-management behaviors for primary care patients who had two or more  visits with outcome ratings documented. | Community Nursing Centers (CNCs) purposefully offer health promotion and primary care services in underserved urban locations where primarily low-income people live, work, play, and pray | **- Integration of Behavioral Health:** Integrating behavioral health services into primary care through collaborative models to enhance access and reduce stigma.  **- Nurse-Led Care:** Nurses led interprofessional teams to provide holistic care for underserved, low-income communities.  **- Use of the Omaha System:** The Omaha System ensured standardized documentation of care.  **- Focus on Social Determinants:** the model addressed social determinants like food insecurity and housing instability. | The primary care team in 2016 comprised one full-time (100 % FTE) clinical nurse specialist/  clinic director, two part-time family nurse practitioners (FNP), totaling 135 % FTEs, and two part-time  registered nurse case managers (i.e., 40 % FTE) from diverse racial backgrounds. | - **Research:** Investigate the long-term effects of integrating behavioral health into nurse-led care, focusing on diverse populations.  - **Policy:**  Support community-based, NLC for providing culturally relevant care.  - Policymakers should fund training programs that incorporate behavioral health skills into nursing education to enhance integrated care. |
| - Kor et al., 2022 [30]  - Hongkong  - NLC  operated by the Nursing Department of a university in Hong Kong  - Community-based nurse-led cognitive  assessment service  - 100 % | cross-sectional design  -223 attendee  -133 completed telephone follow-up  Poor cognitive function  cognitive  assessment service | 1. To explore the clients’ adherence to nursing recommendations  after attending the cognitive assessment service  2. To identify the predictors of client adherence  3. To evaluate the clients’ level of satisfaction with the services of  the NLC | -About 44.1 % of clients receiving a cognitive assessment did not adhere to the nursing recommendations. Those with poorer cognitive functions and lower education levels were found to be less likely to be adherent.  - About 96 % of clients attending the community-based cognitive assessment ser-  vices in an NLC expressed satisfaction with them. | The NLC was operated by the Nursing Department of a university in Hong Kong. | the NLC model, run by nurses specialized in cognitive health and dementia and does not require complex referral procedures.  **Nurse role**  -**Provided physical, functional, cognitive, and mental health assessment** before attending clinic; then, provided health education (disease process and caregiving skills to caregivers).  -**Provided holistic nursing theory to address the emotional, social, and spiritual needs** of the clients and their family members during the counseling session  -**Referred clients for further medical consultations and examinations** if needed (diagnosis, prescribe blood test, and imaging investigations). | Two experienced registered nurses with doctoral degrees who  specialize in gerontology/cognitive health were involved in assessing the clients.  Each practitioner went to 70 hrs. of training in the use of assessment tools, communication, counseling skills, reporting data, handling patients’ emotions, and making referrals; then, practicing at least 5 sessions of cognitive assessment with the chief of service on cognitive impairment care. Their inter-rater reliability was 0.9 or above in every instrument across 5 sessions. | Policy: Community-based cognitive assessment clinics should develop appropriate strategies to improve clients' adherence, especially those with poor cognitive functions and lower education levels.  Future research: further study should investigate why clients with poor cognitive functions and lower education levels are less likely to adhere to nursing recommendations. |
| - Choi et al., 2015 [31]  - Hong Kong, China  - Nurse-Led Continence Care  - 100 % | A case-controlled study | To evaluate whether community-based nurse-led continence care  Interventions are effective in improving outcomes for adult Chinese primary care patients  with lower urinary tract symptoms. | The intervention group had significant improvements in lower urinary tract symptoms severity (*P* < 0.05) and HRQOL (*P* < 0.05). Improvements  in the amount of urine leakage was not significantly different between the two groups.  However, subgroup analysis found that the amount of urinary leakage significantly improved in female subjects in both groups, but not in male subjects. | General Out-Patient Clinic locations are distributed across Hong Kong | **Nurse role**  - **Conducted the consultations, assessment, and provided treatments**, as part of a multi-disciplinary clinic with primary care doctors and nurse assistants.  Interventions include pelvic floor muscle exercise, diet modification, and bladder training  and urethral massage for male patients. | Nurses with a specialty by training in continence care, certified by the Hospital Authority | Future research should examine the long-term effectiveness, adherence, and cost-efficiency of nurse-led continence care programs and compare their outcomes with physician-led models. Policymakers should also consider integrating nurse-led continence services into primary care systems and enhancing nurse training and certification in continence management to ensure sustainable and high-quality care delivery. |
| - Mazza et al., 2023 [32]  - Australia  - NA  - 53.8 % | Protocol RCT  Sequential  32 settings  long-acting reversible contraception (implant) (LARC) and  medical abortion services  GP prescribes the Mifepristone–misoprostol regimen, performs an IUD. | 1. to assess the  effectiveness of a nurse-led model of care (involving  task-sharing, and where appropriate, implant insertion  by nurses and the use of telehealth) in general practice  for improving women’s uptake of LARC and access to  medical abortion in rural and regional areas.  The secondary aims are to:  1. Codesign a nurse-led model of care for LARC provision and medical abortion in rural and regional general practice.  2. Evaluate the implementation of the nurse-led model using realist evaluation approaches.  3. Evaluate the cost-effectiveness of the intervention compared with usual care. | Nurse-led model of care (ORIENT: ImprOving Rural and regIonal accEss to long acting reversible contraceptioN and medical abortion through Nurse-Led Models of Care, Tasksharing and telehealth), supported by task-sharing, clinical upskilling, telehealth, and community-of-practice networks, increased LARC prescribing rates, improved access to medical abortion, and was cost-effective. | General practices located in rural or regional areas across Australia.  Determine practice geographical location by linking practice postcodes to the Modified Monash Model geographical classification that categorizes all areas in Australia into remoteness categories ranging from MM 1 (metropolitan areas) to MM 7 (very remote communities). Aim to include practices that are located in areas rated as MM 2 (ie, live within 20km of a town with 500,000+ people) to MM 7 (ie, very remote). | Counselor and Educator: providing patient education, counseling, and in some cases inserting and removing contraceptive implants.  Care coordinator: linking women with referral pathways (e.g., IUD insertion where GPs are not available) and allied health services. | Two general practitioners, one practicing nurse, and one practice manager. | Policymakers may consider formally expanding the scope of practice for nurses to include LARC insertion, supported by appropriate certification and regulation. The codesign approach in the ORIENT trial should be embedded in reproductive health policy formation to ensure services are acceptable and accessible, especially in rural areas. |
| - Atkinson-Briggs et al., 2023 [33]  - Mooroopna, Victoria, Australia  - Primary health care clinic with nurse-led integrated Diabetes Education  -87.5 % | observational, cross-sectional, single-site  study  172 adults  Participants in the DRS sub-study  *n* = 135 imaged and vision assessed  3 ungradable image sets excluded  *n* = 132 gradable for diabetic retinopathy (DR)  Type 1 diabetes (T1D) or T2D | To describe the prevalence and severity of diabetic retinopathy (DR) and presenting vision level among Indigenous Australian adults with diabetes attending an indigenous primary care clinic in regional Australia. | - The present study demonstrated that a nurse-led model of integrating diabetes eye screening and diabetes education in Indigenous Australian adults with diabetes in a primary care setting is feasible.  - nurse-led model of care integrating diabetes eye screening and education at a single visit was successful at recruiting Indigenous Australian adults with diabetes, screening their vision, and acquiring a high rate of gradable images | - The principal investigator was a nurse, a nationally accredited, credentialled diabetes educator, who was trained in testing vision and retinal imaging. | **Nurse role**  - **Integrated diabetes education with DR** screening, conducted retinal imaging and vision tests, and educated patients using retinal images to promote self-management.  - **Collaborated with patients, healthcare team members** (e.g., GPs, optometrists), **and Indigenous community stakeholders.**  - **Delivered culturally sensitive care,** streamlined services in a single visit, empowered patients through interactive education, and collected clinical data for research. | Nurse, a nationally accredited, credentialled diabetes educator, | Nurse-led model can screen diabetes mellitus patients |
| - Lusaka et al., 2023 [34]  - Liberia  - Community-based clinic (affiliated with a hospital)/ Nurse-led Community-Based, Palliative Care Program  - 80 % | - Qualitative study  - 8 participants  - Palliative Care  Program (for cancer patients) | This is a qualitative process evaluation eliciting the experiences, perspectives, and attitudes of patients with cancer to inform future palliative care program delivery improvement and development. | **5 themes emerged:**  **1) History and disease progression**  • When they received their life-threatening disease diagnoses, some accepted the news quickly, whereas others expressed that they were in a state of denial.  **2) Follow-up clinic services**  • Several participants expressed that the palliative care program services helped minimize the time they spent navigating care provision.  • Patients expressed challenges in reaching and communicating with their palliative care clinicians.  • Despite reported challenges, patients saw that the palliative care team established relationships with health care workers at patients' local health facilities to ensure cross-team communication and streamline care  **3) Psychological distress and its sources**  • Patients reported psychological distress characterized by multiple symptoms, including depression, stress, and anxiety.  **4) Social support**  • Because most of the participants lived in rural areas, experienced poverty, and were dependent on farming for a living, many were reliant on social programs provided by the government. These programs were integral in providing basic hygiene and nutritional supplies for patients as they faced cancer.  **5) Spiritual and cultural beliefs**  • For many patients, disease perceptions were informed by strong spiritual and cultural beliefs, with illnesses and symptoms attributed to witchcraft or supernatural forces. | The definition of community is not clear but this study was conducted in Maryland County, which accounts for Liberia’s highest rates of absolute (84 %), food (71.5 %), and extreme poverty (47.5 %). | **Palliative Care Model**  - Improve the availability, accessibility,  and tracking systems of pain relief medication, follow-up with an appointment, etc.  -Integral in providing basic hygiene and nutritional supplies for patients as they faced cancer. | - Registered nurse (End of Life Nursing Education Consortium Curriculum Training)  - Physician assistant | Future research should evaluate the nurse-led model for implementation in other settings, especially for palliative care, and policies should support its adoption to improve symptom management, accessibility, and continuity of care. |
| - Pandey et al., 2023 [35]  - Canada  - Community partnered  care model  - 80 % | Qualitative  research design  *n* =24  15 clinic providers and 9 community members  Chronic disease | (i) identify key insights and suggest strategies to address healthcare access barriers and (ii) highlight the importance of community engagement and involvement when developing strategies to address Indigenous healthcare needs. | healthcare access in rural Indigenous communities can be improved by developing Outreach clinics that are delivered in partnership with community healthcare teams through community healthcare centers.  A nurse coordinator is crucial in ensuring effective and ongoing communication  between community healthcare teams and urban healthcare teams.  this community-led clinic improves primary care access and chronic disease management in First Nation communities. | Outreach clinical team, a regional health authority research scientist, an epidemiologist, community healthcare providers, community health managers, and peers. | **Community-led clinic**  **- Nurse Coordinator Role:** Liaises between urban providers and local teams, ensuring continuity of care and communication.  **- Holistic Care:** Nurses deliver chronic disease management, screenings, phlebotomy, and education within communities.  **- Community Collaboration:** Nurses engage with leaders, Elders, and staff, integrating traditional healing with Western medicine.  **- Addressing Barriers:** Nurses resolve logistical issues, manage supplies, maintain records, and tackle health literacy and discrimination. | Outreach nurse | Policy: Strengthening cooperation between urban and First Nation healthcare teams can build capacity, ensure timely access to care, and enhance health outcomes. |
| - Fenton et al., 2024 [36]  - USA  - NP-Led Telehealth Practice  - 100 % | - Retrospective design (Using existing data)  - 275  - Mental health care | To understand how an NP-led integrated telemedicine practice can improve access to mental health care in Maryland, particularly in areas with significant mental health needs. | - The NP-led telemedicine practice reached 67 % of all counties in Maryland. - It provided care to 69 % of counties with the highest mental health needs. - The study demonstrates that NP-led telemedicine effectively expands access to mental health care. - It highlights the potential for targeting underserved areas lacking adequate mental health services. | Using Telehealth to provide mental health care in counties in Maryland, USA. | **NP-led integrated telemedicine practice focused on mental health care**  **- Full Practice Authority for NP**: Allowing NPs to provide comprehensive mental health services without physician oversight.  **- Telemedicine Integration**: Utilizing telehealth technology to enhance access to care, particularly in underserved areas.  **- Patient-Centered Approach**: Prioritizing patient experience and convenience by reducing transportation barriers and improving access to trained mental health providers.  **- Focus on Health Equity**: Addressing disparities in mental health care access by considering factors such as Internet connectivity and resource availability in communities.  **- Collaborative Care**: Encouraging partnerships with community organizations and cross-training among providers to improve service delivery in high-need areas. | - Psychiatric NP  - N/A | Research: Future study should evaluate the long-term effectiveness of NP-led telemedicine models in underserved areas and strategies to improve technology access.  Policy-makers should prioritize funding for telehealth initiatives, promote provider cross-training, and establish community partnerships to address care gaps. Ongoing assessment of service quality and patient outcomes is essential for continuous improvement. |

*Note*: NLC = Nurse-Led Clinic. PHC = Primary Health Care. NP = Nurse Practitioner. GP = General Practitioner. RCT = Randomized Controlled Trial. IUD = Intrauterine Device. BH = Behavioral Health. PATH = Providing Access to Healthcare. PHQ-9 = Patient Health Questionnaire-9. GAD-7 = Generalized Anxiety Disorder-7. PrEP = Pre-Exposure Prophylaxis. RN = Registered Nurse. STI = Sexually Transmitted Infection. T1D = Type 1 Diabetes. T2D = Type 2 Diabetes. CNC = Community Nursing Center. FTE = Full-Time Equivalent. FNP = Family Nurse Practitioner. HRQoL = Health-Related Quality of Life.

Appendix C. Model of care in nurse-led clinics in the community

| References  (*n* = 13) | Model of care in nurse-led clinics in the community | | | | | |
| --- | --- | --- | --- | --- | --- | --- |
|  | 1. Innovative cognitive care | 2. Integrated multidisciplinary care | 3. Community-driven underserved population care | 4. Reproductive and women’s health innovation | 5. Palliative care model | 6. Behavioral health integration |
| Gagné, et al., 2021 [24] |  | **X** |  |  |  |  |
| Yan, et al., 2024 [25] | **X** |  |  |  |  |  |
| Tominc, et al., 2023 [26] |  | **X** | **X** |  |  |  |
| Talley, et al., 2021 [27] |  |  |  |  |  | **X** |
| O'Byrne, et al., 2021 [28] |  | **X** |  |  |  |  |
| Holt, et al., 2020 [29] |  |  |  |  |  | **X** |
| Kor, et al., 2022 [30] | **X** |  |  |  |  |  |
| Choi, et al., 2015 [31] |  | **X** |  |  |  |  |
| Mazza, et al., 2023 [32] |  |  |  | **X** |  |  |
| Atkinson‐Briggs, et al., 2023 [33] |  |  | **X** |  |  |  |
| Lusaka, et al., 2023 [34] |  |  |  |  | **X** |  |
| Pandey, et al., 2023 [35] |  | **X** | **X** |  |  |  |
| Fenton, et al., 2024 [36] |  | **X** |  |  |  |  |
| Number (%) | 2 (15.38 %) | 6 (46.14 %) | 3 (23.07 %) | 1 (7.69 %) | 1 (7.69 %) | 2 (15.38%) |
